# Supplementary material for: YAP inhibition enhances the differentiation of functional stem cell-derived insulin-producing β cells
Source: Nat Commun. 2019 Apr 1;10:1464. doi: 10.1038/s41467-019-09404-6 (PMC6443737; doi:10.1038/s41467-019-09404-6)
Supplement: Supplementary file 1 — Supplementary Information [file 41467_2019_9404_MOESM1_ESM.pdf]

Supplementary Information

YAP inhibition enhances the differentiation of functional stem cell-derived insulin-producing β cells

Rosado-Olivieri et al.

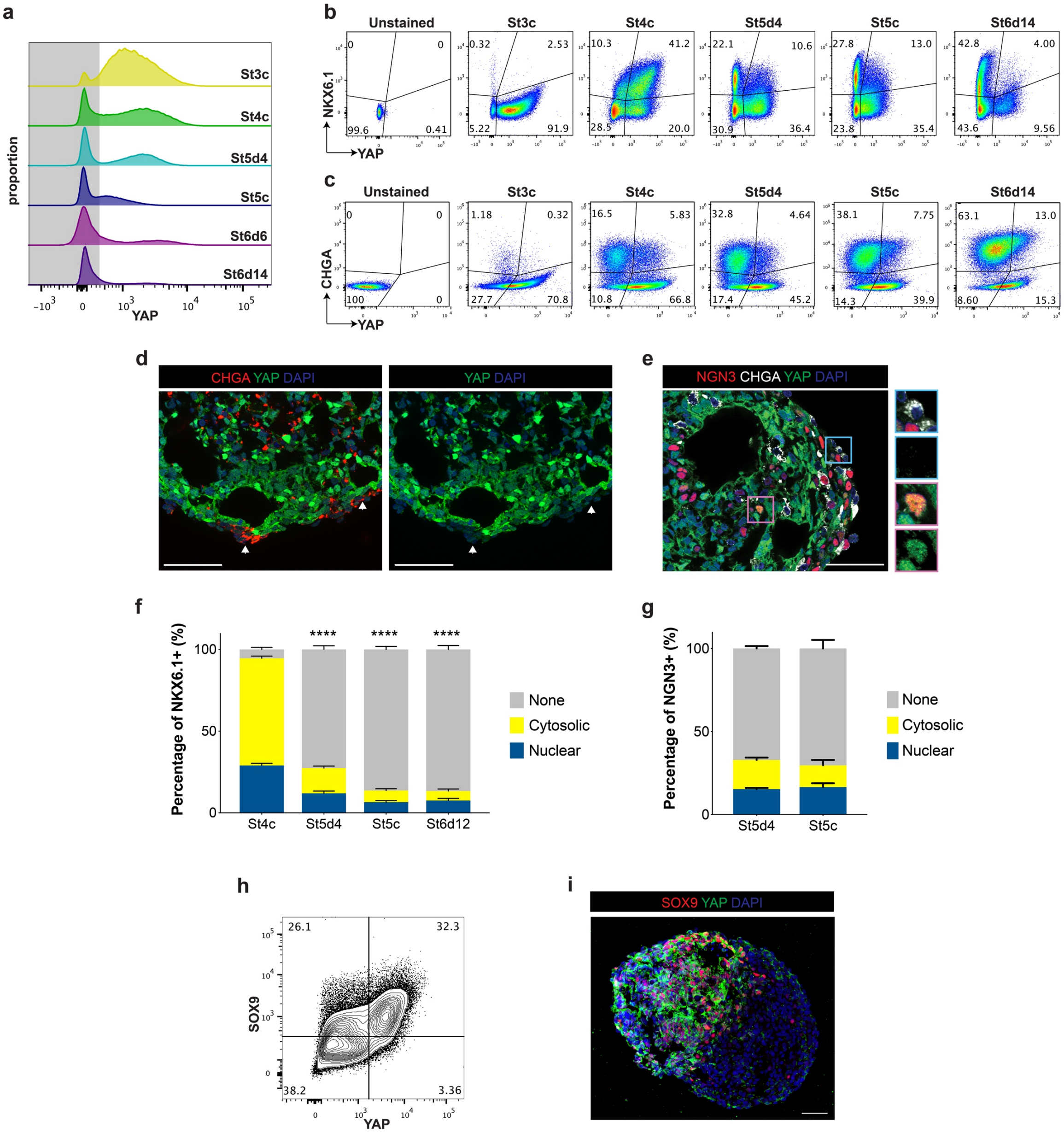

Supplementary Figure 1: YAP expression in progenitor cells during endocrine differentiation. (a) Histogram plots of YAP expression of cells collected from stage 3 through stage 6 of β cell differentiation, assayed by flow cytometry. Highlighted area in grey denotes gate on YAP-negative cells. **(b-c)** Flow cytometry analysis of co-expression of YAP with NKX6.1 (b) and CHGA (c) at multiple stages of β cell differentiation. **(d)** Immunohistological analysis of YAP and CHGA expression in pancreatic progenitors (end of stage 4). Arrows indicate a downregulated expression of YAP in CHGA+ endocrine cells. **(e)** Immunofluorescent micrographs of YAP, NGN3, and CHGA in differentiating endocrine progenitors (stage 5, day 3). **(f-g)** Immunohistological analysis of nuclear, cytoplasmic or downregulated expression of YAP in NKX6.1+ (f) and NGN3+ cells (g) at stage 4, 5 and 6. **(h-i)** Co-expression of YAP and SOX9 in progenitors after endocrine induction as assessed by flow cytometry (e) and immunohistochemistry (f). Scale bar: 50 μm. Data represent mean ±SEM, ****p<0.0001, two-sided student's t-test (n=3 biologically independent samples per group). St4c: stage 4 complete, St5c: stage 5 complete.

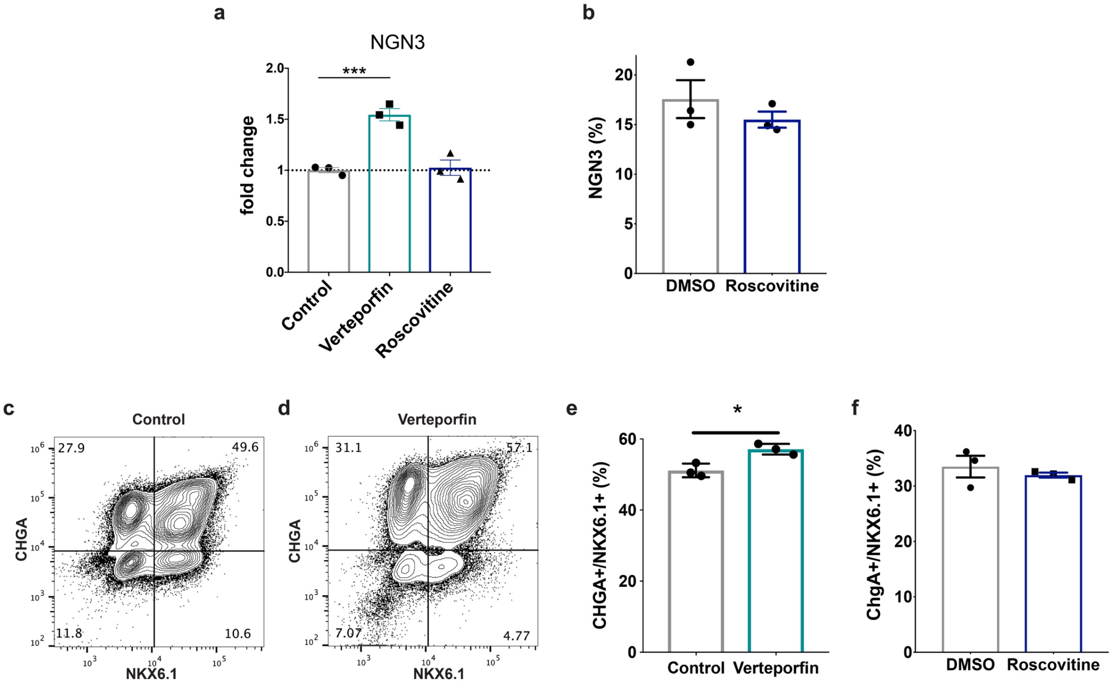

Supplementary Figure 2: Cell cycle inhibition does not enhance endocrine differentiation. **(a)** qPCR analysis of NGN3 expression of verteporfin and roscovitine-treated progenitors at stage 5, day 4. qPCR values normalized to the average expression value of control samples. **(b)** Proportion of NGN3+ cells in roscovitine-treated differentiating MPPs as assayed by flow cytometry at stage 5, day 4. **(c-f)** Flow cytometry analysis of CHGA and NKX6.1 expression upon completion of the endocrine specification stage (stage 5) and quantification of the proportion of CHGA+/NKX6.1+ cells in control, verteporfin- and roscovitine treated differentiations. Data represent mean ±SEM, *$p<0.05$, ****$p<0.0001$, two-sided student's t-test (n=3 biologically independent samples per group).

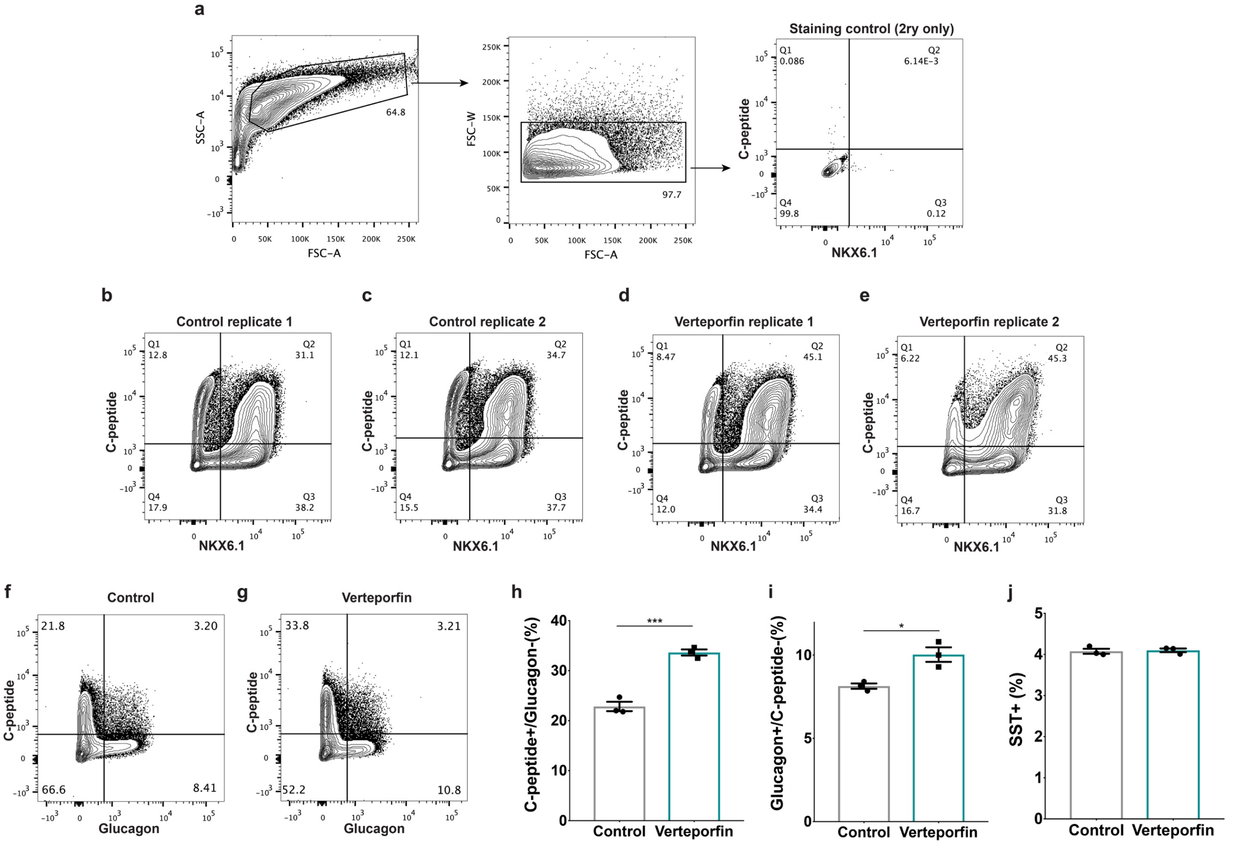

Supplementary Figure 3: Enhanced endocrine differentiation upon YAP inhibition. **(a)** Gating strategy and staining control for b-e and Fig. 3e-f. **(b-e)** Staining control and replicate data for control and verteporfin-treated differentiations collected at the end of stage 6 differentiation (stage 6 day 14). **(f-j)** Flow cytometry analysis of C-peptide, Glucagon and Somatostatin expression and quantification of the proportion of monohormonal endocrine cells in control and verteporfin-treated differentiations. Data represent mean ±SEM, *p<0.05, ***p<0.001, two-sided student's t-test (n=3 biologically independent samples per group).

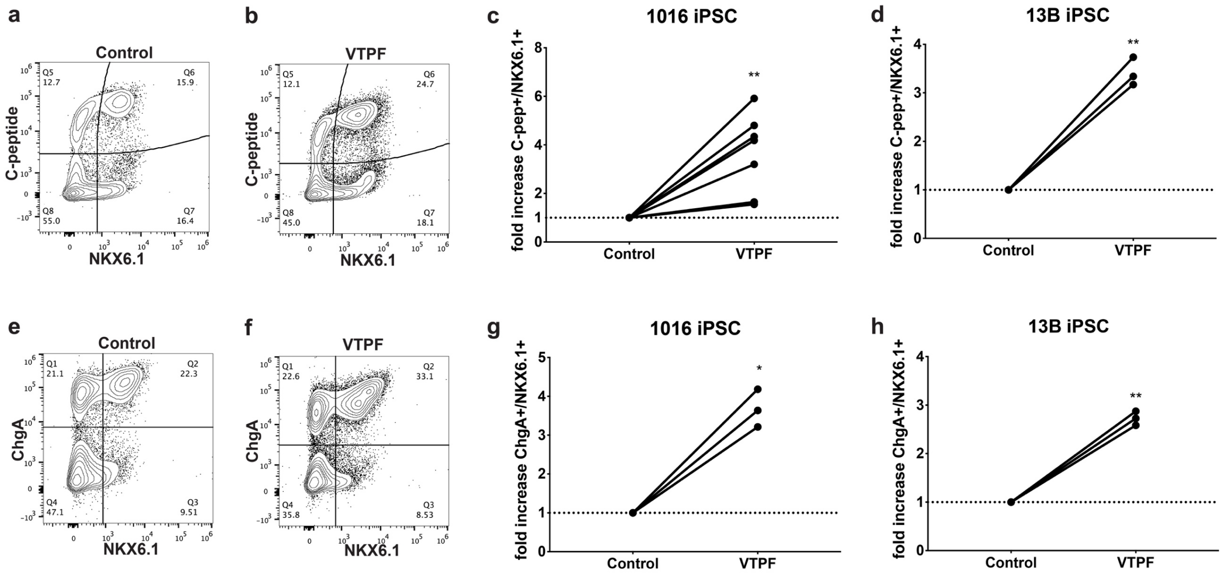

Supplementary Figure 4: YAP inhibition enhances endocrine differentiation of multiple hPSC cell lines. **(a-d)** Flow cytometry and quantification of C-peptide and NKX6.1 expression of control and verteporfin-treated differentiations performed with the 1016 and 13B iPSC cell lines collected at the end of stage 6 (stage 6 day 14). **(e-h)** Flow cytometry and quantification of CHGA and NKX6.1 expression at the end of stage 6 (stage 6 day 14). Data presented as a fold increase over stage-matched control differentiations. *$p<0.05$, **$p<0.01$, two-sided t-test on log fold changes (n=3 biologically independent samples per group).

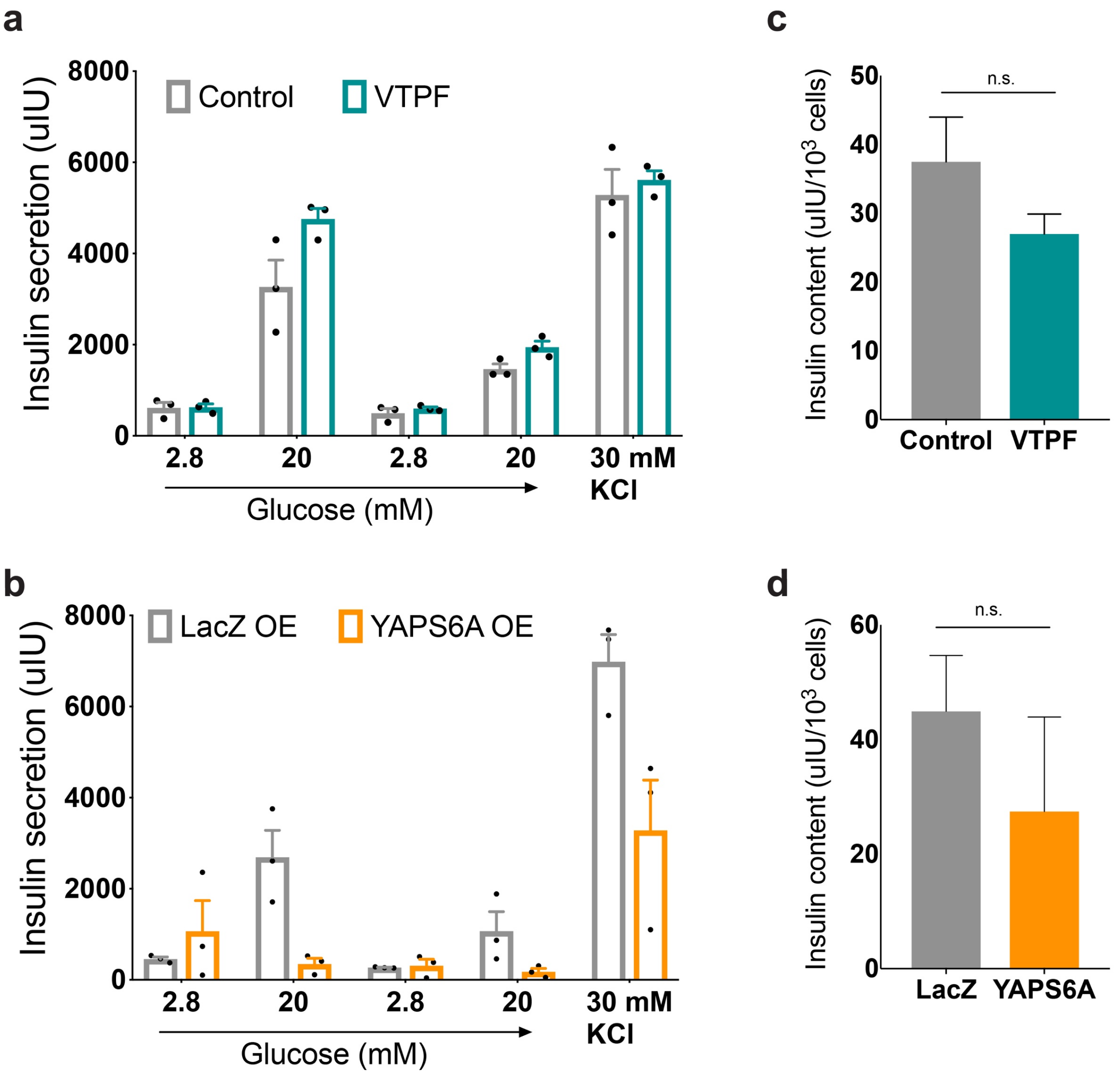

Supplementary Figure 5: Insulin secretion and insulin content measurements of SC-β cells. (a-b) Raw insulin secretion values for stage-matched control, verteporfin-treated and YAPS6A-overexpressing β cells during sequential stimulations with low and high glucose, and KCl depolarization. **(c-d)** Insulin content measurements normalized to total number of cells of stage matched SC-β cells. Data represent mean ± SEM, two-sided student's t-test (n=3 biologically independent samples per group). n.s.: not-significant.

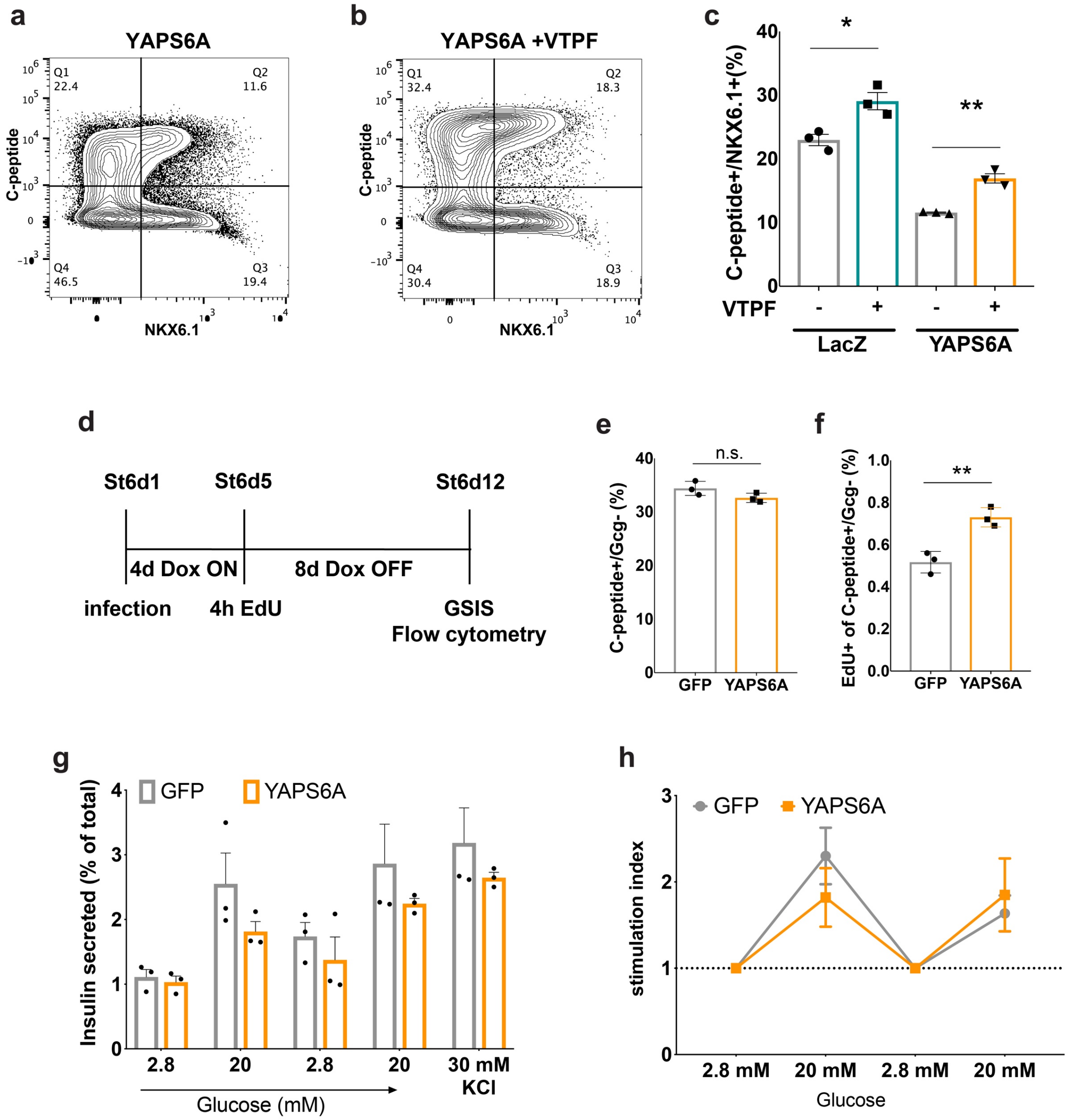

Supplementary Figure 6: YAPS6A inhibits SC-β cell differentiation. (a-c) Flow cytometry analysis and quantification of C-peptide and NKX6.1 expression of LacZ or YAPS6A-overexpressing SC-β cells differentiated in the presence of verteporfin and collected at the end of stage 6 differentiation (stage 6 day 14). **(d)** Experimental design for e-h. Expression of YAPS6A and GFP was transiently induced with doxycycline during the first 4 days of stage 6 (Dox ON), followed by 8 days with no doxycycline added (Dox OFF). **(e-f)** Flow cytometry analysis and quantification of C-peptide and NKX6.1 expression as well as EdU staining of SC-β cells collected at stage 6 day 12 as outlined in d. EdU pulse was performed for 4 hours and 4 days after YAPS6A/GFP induction. **(g-h)** Insulin secretion during sequential stimulations with low and high glucose, and KCl depolarization of SC-β cells after a transient overexpression of YAPS6A or GFP as outlined in d. Data represent mean ± SEM. *p<0.05, **p<0.01, two-sided student's t-test (n=3 biologically independent samples per group). n.s.: non-significant, DOX: doxycycline.

Supplementary Table 1: List of taqman probes used for qPCR analysis.

Target gene	Probe id
GAPDH	Hs02786624_g1
YAP1	Hs00902712_g1
CYR61	Hs00155479_m1
CTGF	Hs00170014_m1
GLI2	Hs00170014_m1
NEUROG3	Hs01875204_s1
