# Supplementary material for: YAP inhibition enhances the differentiation of functional stem cell-derived insulin-producing β cells
Source: Nat Commun. 2019 Apr 1;10:1464. doi: 10.1038/s41467-019-09404-6 (PMC6443737; doi:10.1038/s41467-019-09404-6)
Supplement: Supplementary file 3 — Reporting Summary [file 41467_2019_9404_MOESM3_ESM.pdf]

nature research

Corresponding author(s): Douglas A. Melton

Last updated by author(s): Feb 7, 2019

Reporting Summary

Nature Research wishes to improve the reproducibility of the work that we publish. This form provides structure for consistency and transparency in reporting. For further information on Nature Research policies, see Authors & Referees and the Editorial Policy Checklist.

Statistics

For all statistical analyses, confirm that the following items are present in the figure legend, table legend, main text, or Methods section.

n/a	Confirmed	
☐	☒	The exact sample size (*n*) for each experimental group/condition, given as a discrete number and unit of measurement
☐	☒	A statement on whether measurements were taken from distinct samples or whether the same sample was measured repeatedly
☐	☒	The statistical test(s) used AND whether they are one- or two-sided<br>*Only common tests should be described solely by name; describe more complex techniques in the Methods section.*
☐	☒	A description of all covariates tested
☐	☒	A description of any assumptions or corrections, such as tests of normality and adjustment for multiple comparisons
☐	☒	A full description of the statistical parameters including central tendency (e.g. means) or other basic estimates (e.g. regression coefficient) AND variation (e.g. standard deviation) or associated estimates of uncertainty (e.g. confidence intervals)
☐	☒	For null hypothesis testing, the test statistic (e.g. *F*, *t*, *r*) with confidence intervals, effect sizes, degrees of freedom and *P* value noted<br>*Give P values as exact values whenever suitable.*
☒	☐	For Bayesian analysis, information on the choice of priors and Markov chain Monte Carlo settings
☒	☐	For hierarchical and complex designs, identification of the appropriate level for tests and full reporting of outcomes
☒	☐	Estimates of effect sizes (e.g. Cohen's *d*, Pearson's *r*), indicating how they were calculated

Our web collection on statistics for biologists contains articles on many of the points above.

Software and code

Policy information about availability of computer code

Data collection	BD FACSDiva software was used for flow cytometry data collection using BD LSRII . SDS 2.3 software was used for qPCR data collection using machine ABI 7900HT PCR .
Data analysis	FlowJo v10, R v3.5.1 and GraphPad Prism 8 were used for data analysis.

For manuscripts utilizing custom algorithms or software that are central to the research but not yet described in published literature, software must be made available to editors/reviewers. We strongly encourage code deposition in a community repository (e.g. GitHub). See the Nature Research guidelines for submitting code & software for further information.

Data

Policy information about availability of data

All manuscripts must include a data availability statement. This statement should provide the following information, where applicable:

- Accession codes, unique identifiers, or web links for publicly available datasets
- A list of figures that have associated raw data
- A description of any restrictions on data availability

-There are no restrictions on data availability.
- Figures with raw data: Supplementary Figure 5

Field-specific reporting

Please select the one below that is the best fit for your research. If you are not sure, read the appropriate sections before making your selection.

☒ Life sciences ☐ Behavioural & social sciences ☐ Ecological, evolutionary & environmental sciences

For a reference copy of the document with all sections, see nature.com/documents/nr-reporting-summary-flat.pdf

Life sciences study design

All studies must disclose on these points even when the disclosure is negative.

Sample size	No statistical analysis was used to predetermine sample size. Sample size was determined based on prebious studies on the same subject. Sample size was at least n=3 biological replicates for all the experiments. These are shown on the figures with dot plots overlaying bar graphs.
Data exclusions	No data were excluded.
Replication	3 or more biological replicates were considered for the analysis using stage-matched controls as a reference. All the experiments and results included in this study were confirmed with three or more independent experimental repeats. Statistical analysis was performed by using unpaired two-sided t tests unless stated otherwise. All attempts to replicate the experiments were successful.
Randomization	There was no randomization for the experiments as it did not involve clinical studies.
Blinding	Investigators were blinded to samples collected from in vivo transplantation studies and immunostaining analysis. Investigators were not blinded to group allocation as phenotypes were identifiable during the analysis.

Reporting for specific materials, systems and methods

We require information from authors about some types of materials, experimental systems and methods used in many studies. Here, indicate whether each material, system or method listed is relevant to your study. If you are not sure if a list item applies to your research, read the appropriate section before selecting a response.

Materials & experimental systems

n/a	Involved in the study	
☐	☒	Antibodies
☐	☒	Eukaryotic cell lines
☒	☐	Palaeontology
☐	☒	Animals and other organisms
☒	☐	Human research participants
☒	☐	Clinical data

Methods

n/a	Involved in the study	
☒	☐	ChIP-seq
☐	☒	Flow cytometry
☒	☐	MRI-based neuroimaging

Antibodies

Antibodies used	The antibodies used in the study were the following: rabbit anti-YAP (Cell Signaling Technology; 14074S), mouse anti-YAP (Abnova; 89106308), rabbit anti-SOX9 (Cell Marque; AC-0284RUO), rat anti-C-peptide (Developmental Studies Hybridoma Bank; GN-ID4), mouse anti-NKX6.1 (Developmental Studies Hybridoma Bank; F55A12-supernatant), rabbit anti-Ki67 (Abcam; ab16667), sheep anti-NGN3 (R&D systems; AF3444), goat anti-PDX1 (R&D systems; AF2419), rabbit anti-CHGA (Novus Biologicals; NB120-15160), mouse anti-CHGA (Santa Cruz; sc-393941), mouse anti-Glucagon (Abcam; ab82270), mouse anti-Somatostatin (Santa Cruz; sc-55565), rabbit anti-cleaved Caspase-3 (Cell Signaling; 9661) and mouse anti-PCNA (Millipore; NA03).
Validation	The antibodies have been validated as noted on manufacturer's website. Additional validation was performed by using negative controls and control tissue samples. The expression patterns observed with all the antibodies included in our paper display stage-specific expression by immunostaining and flow cytometry.

Eukaryotic cell lines

Policy information about cell lines

Cell line source(s)	HUES8 and 1016 cell lines were obtained from Human Embryonic Stem Cell Facility and iPS Core Facility of the Harvard Stem Cell Institute. 13B iPSC cell line was obtained from University of Massachusetts Medical School.
Authentication	None of the cell lines were authenticated.

Mycoplasma contamination	All cell lines tested negative for mycoplasma contamination.
Commonly misidentified lines (See ICLAC register)	No cell lines used in this study were found in the ICLAC database.

Animals and other organisms

Policy information about studies involving animals; ARRIVE guidelines recommended for reporting animal research

Laboratory animals	Immunodeficient SCID-beige mice (Mus musculus).
Wild animals	The study did not involve wild animals.
Field-collected samples	The study did not involve field-collected samples.
Ethics oversight	All animal experiments were performed in accordance with Harvard University International Animal Care and Use Committee (IACUC) regulations.

Note that full information on the approval of the study protocol must also be provided in the manuscript.

Flow Cytometry

Plots

Confirm that:

☒ The axis labels state the marker and fluorochrome used (e.g. CD4-FITC).

☒ The axis scales are clearly visible. Include numbers along axes only for bottom left plot of group (a 'group' is an analysis of identical markers).

☒ All plots are contour plots with outliers or pseudocolor plots.

☒ A numerical value for number of cells or percentage (with statistics) is provided.

Methodology

Sample preparation	Differentiated cell clusters were dispersed into a single-cell suspension with TrypLE (Life Technologies) at RT and fixed with 4% paraformaldehyde at 4°C.
Instrument	Data collection was performed with LSR II flow cytometers (BD Biosciences).
Software	BD FACSDiva software was used for data collection and FlowJo v10 was used for data analysis.
Cell population abundance	Flow cytometry was used for marker expression analysis of fixed samples. No cell sorting was performed.
Gating strategy	Cells were first gated based on light scatter properties based on cell size (FSC-A) and granularity (SSC-A), and width parameter on foward scatter was used to gate out doublets. For marker expression, negative cells were gated based on secondary only staining control. Cells expressing relevant markers were classified based on unstained negative cells.

☒ Tick this box to confirm that a figure exemplifying the gating strategy is provided in the Supplementary Information.
